# Prognostic and clinicopathological value of Twist expression in breast cancer: A meta-analysis

**DOI:** 10.1371/journal.pone.0186191

**Published:** 2017-10-09

**Authors:** Weiqiang Qiao, Zhiqiang Jia, Heyang Liu, Qipeng Liu, Ting Zhang, Wanying Guo, Peng Li, Miao Deng, Sanqiang Li

**Affiliations:** 1 Department of Breast Surgery, The First Affiliated Hospital, and College of Clinical Medicine of Henan University of Science and Technology, Luoyang, China; 2 Department of Spinal Surgery, The Second Affiliated Hospital of Henan University of Science and Technology, Luoyang, China; 3 Department of Oncology, The First Affiliated Hospital, and College of Clinical Medicine of Henan University of Science and Technology, Luoyang, China; 4 The Molecular Medicine Key Laboratory of Liver Injury and Repair, Medical College, Henan University of Science and Technology, Luoyang, China; Fondazione IRCCS Istituto Nazionale dei Tumori, ITALY

## Abstract

**Background:**

Despite initial indications that the transcription factor Twist could be used as a breast cancer prognostic marker, there still exists some controversy about its reliability. Thus, the aim of the present study was to assess the relationship between Twist expression and prognosis in breast carcinoma.

**Materials and methods:**

We identified eligible studies that reported an association between Twist expression and breast cancer prognosis by searching the literature in PubMed, Embase, the Cochrane Library, and Web of Science databases, through June 5, 2017. Studies investigating Twist protein or mRNA expression as well as reporting survival data in breast cancer were included. The pooled hazard ratio (HR) and odds radio (OR) with a 95% confidence interval (95% CI) were used to estimate associations.

**Results:**

A total of 2,671 patients from seven included studies were assessed, and the data indicated that increased Twist expression significantly correlated with poor overall survival (OS) (HR, 1.15; 95% CI, 1.00–1.33; P = 0.04) in breast cancer. In addition, we also observed a significant correlation of elevated Twist expression with larger tumor size (OR, 1.92; 95% CI, 1.31–2.81; P = 0.0009), lymph node involvement (OR, 3.81; 95% CI, 1.16–12.54; P = 0.03), higher nuclear grade (OR, 1.45; 95% CI, 1.06–2.00; P = 0.02), and positive human epidermal growth factor receptor 2 (HER2) status (OR, 1.49; 95% CI, 1.06–2.09; P = 0.02). However, no correlation between Twist expression and disease-free survival (DFS), age, estrogen receptor (ER) status, and progesterone receptor (PR) status was observed.

**Conclusions:**

Our results demonstrate that Twist over-expression is a statistically significant indicator of OS in breast cancer. In addition, our meta-analysis shows that increased Twist expression is significantly associated with larger tumor size, lymph node involvement, higher nuclear grade, and positive HER2 status.

## Introduction

Breast cancer incidence is high not only in Chinese women but also worldwide, and thus is ranked as one of the most common cancers[1, 2]. Breast cancer is categorized into different subtypes based on the expression of various biomarkers, including estrogen receptor (ER), progesterone receptor (PR), human epidermal growth factor receptor 2 (HER2), and Ki67. Expression of these markers plays a vital role in deciding the fundamental therapeutic strategy[3]. As a result, the mortality rate associated with breast cancer has reduced due to significant progress in early diagnosis and development of multiple treatment options. However, a significant percentage of the patient population still fails to respond to these already developed therapies, and many patients develop metastasis, relapse, or display therapeutic resistance. Notable among them is triple-negative breast cancer (TNBC) subtype. Thus, there is an essential need to identify additional novel molecular biomarkers that have the potential to predict therapeutic value across multiple subtypes and serve as therapeutic targets.

Twist is a basic helix-loop-helix (bHLH) transcription factor that has been previously implicated in cell lineage determination and differentiation during embryogenesis. In recent years, Twist has also been shown to contribute to carcinogenesis through triggering epithelial to mesenchymal transition (EMT) and downregulating E-cadherin expression, thereby influencing tumor invasion, metastasis, adverse prognosis, and drug resistance in multiple tumors[4, 5]. Previous studies have demonstrated that Twist interfered the ARF-p53 pathway to prevent c-myc-induced apoptosis and the anti-apoptotic character of Twist was considered the reason for metastatic process[6–9]. Twist expression has been significantly associated with invasion and metastasis of various cancers, including breast cancer[10], non-small cell lung cancer[11], prostate cancer[12], gastric cancer[13], melanoma[14], Sezary syndrome[15], osteosarcoma[16], and hepatocarcinoma[17]. Some studies have indicated that increased Twist expression correlated with worse breast cancer prognosis[18, 19], while other studies showed opposite results[20]. Therefore, to further clarify the prognostic value of Twist in breast cancer, we conducted a new meta-analysis to estimate the association between Twist expression and survival outcomes in breast cancer. In addition, we also assessed the correlation of Twist with clinicopathological features of breast carcinoma.

## Materials and methods

### Search strategy

Eligible studies through June 5, 2017 were identified using PubMed, Embase, the Cochrane Library, and Web of Science databases. The following MeSH terms were used to search relevant articles: “breast neoplasms” and/or “breast cancer”, and/or “Twist”, and/or “prognosis”. Moreover, the reference lists of eligible studies were further searched manually to identify additional relevant studies.

### Inclusion and exclusion criteria

Studies were included in our meta-analysis based on the following criteria: (1) all studies provided information about survival outcome in breast cancer; (2) Twist expression was analyzed in all breast cancer patients; and (3) the hazard ratio (HR) with 95% confidence interval (CI) was either available or sufficient information was available to indirectly estimate it. Studies were excluded from the meta-analysis if they were either (1) duplicate studies, (2) animal or cell studies, (3) HR information was not available and could not be extracted from a Kaplan-Meier curve, or (4) if they were reviews, letters, or only case reports.

### Data extraction

Data extraction from the eligible studies was performed independently by two authors (Weiqiang Qiao and Heyang Liu). The extracted information included: author of publication, year, country, number of patients, age, time of follow-up, clinical outcome, survival analysis, Twist expression, detection method, its cut-off values, antibody, proportion of tumors with Twist over-expression, and correlation between Twist mRNA and protein levels. The HR information was directly recorded if present, or extracted from the Kaplan-Meier curves using Engauge Digitizer Version 4.1 (http://markummitchell.github.io/engauge-digitizer/) software. The quality of the studies was assessed using the modified Newcastle Ottawa Scale (NOS)[21], which classified the studies into eight categories and scored them. A score of 9 represented a maximum score; however, a score of 7 or higher indicated high quality.

### Statistical analysis

The complete meta-analysis was performed using Preferred Reporting Items for Systematic Reviews and Meta-analyses (PRISMA) guideline[22] (S1 File). In studies where HR value was not available, HR was calculated from Kaplan-Meier curves, as suggested by Tierney *et al*.[23]. The Cochran Q test and I^2^ statistics were applied to detect heterogeneity. A P value of < 0.05 and I^2^ value of > 50% represented strong heterogeneity[24]. The fixed effects model was applied for meta-analysis if very low or no heterogeneity was observed. In contrast, the random effects model was used when notable heterogeneity existed between different studies[25]. In addition, sensitivity analysis was also conducted to assess the robustness of the data. The Meta-regression analysis was applied to estimate the sources of heterogeneity. Moreover, potential publication bias was tested using Begg’s test[26]. Overall, all analyses were conducted using Review Manager version 5.3 (Cochrane Collaboration, Copenhagen, Denmark), and STATA version 14.0 (Stata Corporation, TX, USA) software. All statistical tests were two-sided, and a P value of < 0.05 represented a statistically significant difference.

## Results

### Identification of relevant studies

**Fig 1:**
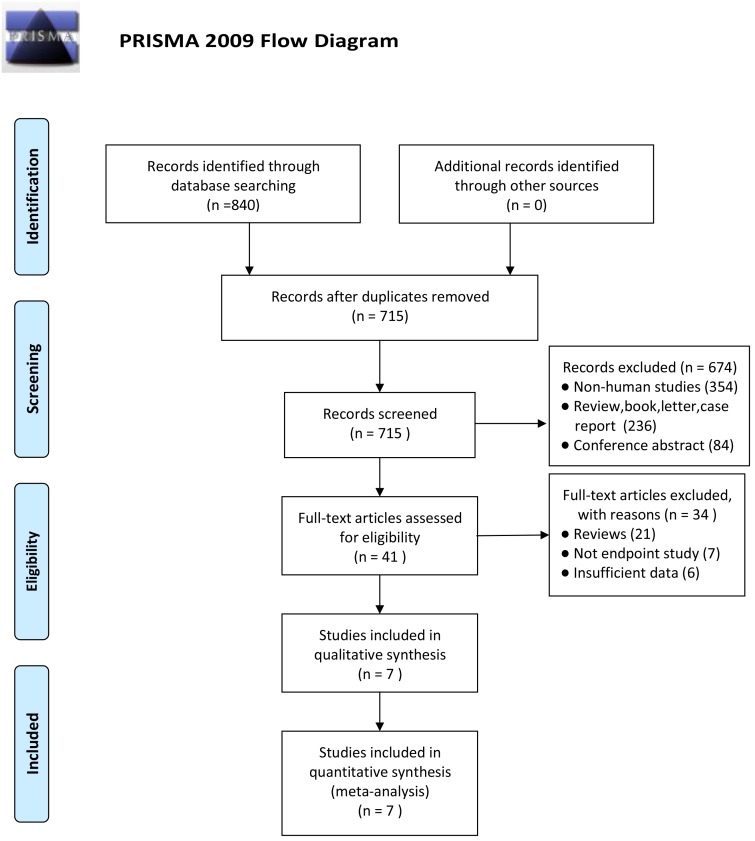
Flow chart of study selection process.

A total of 840 initial studies were identified based on the search strategy (S2 File). Among these, 125 duplicates studies were excluded. After screening the titles and abstracts of the remaining studies, 674 articles were further excluded, thus leaving 41 studies for full review. Based on the exclusion criteria, another 34 studies (21 review articles, 7 with no endpoint, and 6 with insufficient data) were removed. The remaining 7 eligible studies were included in our meta-analysis, which included 2,671 patients[18–20, 27–30]. The flow chart for study selection is outlined in Fig 1.

### Characteristics of eligible studies

The primary characteristics of all the included studies are shown in Tables 1 and 2. All of these articles were primarily published between the years 2011 and 2015. The included studies used different techniques to measure Twist expression. Seven studies analyzed Twist expression using immunochemical (IHC) staining, while 3 studies used reverse transcriptase polymerase chain reaction (RT-PCR). There was some inconsistency regarding Twist expression, as a few studies analyzed Twist, while others specifically measured Twsit1 levels. In addition, we also observed variation in the cut-off values for Twist expression among different studies. Overall, all included studies tested the correlation between Twist expression and breast tumor prognosis. Importantly, all the included studies were observational studies, and thus their quality was assessed using the NOS criteria. Our analysis revealed that all studies were of high quality with a score of ≥ 7 score, as shown in S1 Table.

**Table 1:** Characteristics of eligible studies.

Publication	Year	Country	Cancer subtype	No. of patients	Median age (years)	Median follow-up (years)	Outcome	Survival analysis	NOS (score)
Markiewicz	2012	Poland	II-III BC	36	56.5	4.2	DFS, OS	multivariate	8
Montserrat	2011	Spain	invasive BC	76	67	4.5	DFS, OS	multivariate	8
Riaz	2012	Netherlands	primary BC	1427	55	8.7	DFS, OS	multivariate	8
Soini	2011	Finland	invasive BC	387	NR	NR	OS	univariate	7
Xu	2014	China	primary BC	137	NR	5	DFS, OS	multivariate	8
Zhang	2015	China	invasive BC	408	50	1.3	DFS, OS	univariate	7
Zhao	2013	China	primary BC	200	50	NR	OS	univariate	7

BC, breast cancer; NR, not reported; DFS, disease-free survival; OS, overall survival; NOS, Newcastle Ottawa Scale

**Table 2:** Methods of quantitative Twist measurement of eligible studies.

Publication	Year	Twist phenotype	Detection method	Twist expression	Antibody	Cut-off value (low/high level)	High Twist expression	Correlation between Twist mRNA and protein levels	
Markiewicz	2012	Twist1	RT-PCR, IHC	mRNA, protein	anti-Twist1 (ab50581, Abcam)	NR	66%(29/44)	kappa coefficient	P = 0.002
Montserrat	2011	Twist	RT-PCR, IHC	mRNA, protein	anti-Twist polyclonal antibodies	low(≤10%),high (>10%)	16%(12/76)	Spearman rank test	P = 0.009
Riaz	2012	Twist1	RT-PCR, IHC	mRNA, protein	envision mouse kit, DAKO	NR	NR	Spearman rank test	P < 0.004
Soini	2011	Twist	IHC	protein	mouse monoclonal anti-twist antibody	low(≤5%),high (>5%)	35%(135/387)	NR	NR
Xu	2014	Twist1	IHC	protein	anti-Twist1 (ab50887, Abcam, MA)	high (staining score≥3)	46.7%(64/137)	NR	NR
Zhang	2015	Twist	IHC	protein	mouse monoclonal antibody	NR	53%(220/408)	NR	NR
Zhao	2013	Twist	IHC	protein	anti-Twist polyclonal antibody	high (staining score≥6)	75.5%(151/200)	NR	NR

NR, not reported; RT-PCR, reverse transcriptase polymerase chain reaction; IHC, immunohistochemistry

### Correlation analysis of Twist expression with disease free survival & overall survival

**Fig 2:**
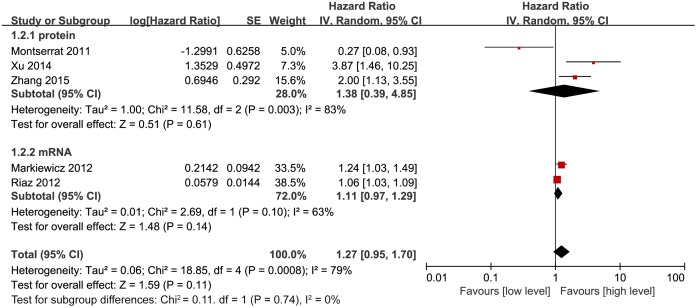
Forest plot depicting association between Twist expression and DFS in breast cancer.

Among the 7 included studies, 5 estimated the correlation between Twist expression and disease-free survival (DFS) in breast cancer patients. Our meta-analysis used the random effect model due to the high heterogeneity (P = 0.0008, I^2^ = 79%) between studies and revealed that Twist expression did not correlate with DFS in breast cancer patients (HR, 1.27; 95% CI, 0.95–1.70; P = 0.11; Fig 2). Next, we assessed this correlation based on Twist expression (mRNA or protein) stratification, but this analysis also did not show any association between Twist expression and DFS. The HR value based on Twist protein expression was 1.38 (95% CI, 0.39–4.85; P = 0.61; Fig 2), while the HR for mRNA levels was 1.11 (95% CI, 0.97–1.29; P = 0.14; Fig 2).

**Fig 3:**
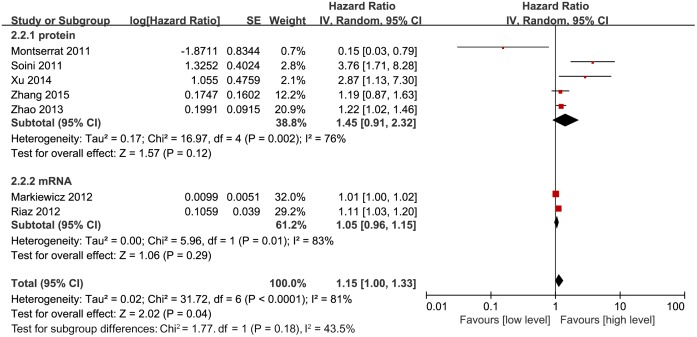
Forest plot depicting association between Twist expression and OS in breast cancer.

Similarly, we also assessed the correlation of Twist expression with overall survival (OS). All 7 studies had data about pooled HRs for OS. Interestingly, increased Twist expression was significantly associated with worse OS (HR, 1.15; 95% CI, 1.00–1.33; P = 0.04; Fig 3). This analysis was performed using random effect model due to significant heterogeneity (P < 0.0001, I^2^ = 81%) between the studies. To understand the reasons of high heterogeneity, we performed further subgroup analyses. Surprisingly, there was no significant correlation for either Twist protein (HR, 1.45; 95% CI, 0.91–2.32; P = 0.12; Fig 3) or mRNA (HR, 1.05; 95% CI, 0.96–1.15; P = 0.29; Fig 3) levels with OS.

### Correlation between Twist expression and other breast cancer clinical parameters

**Fig 4:**
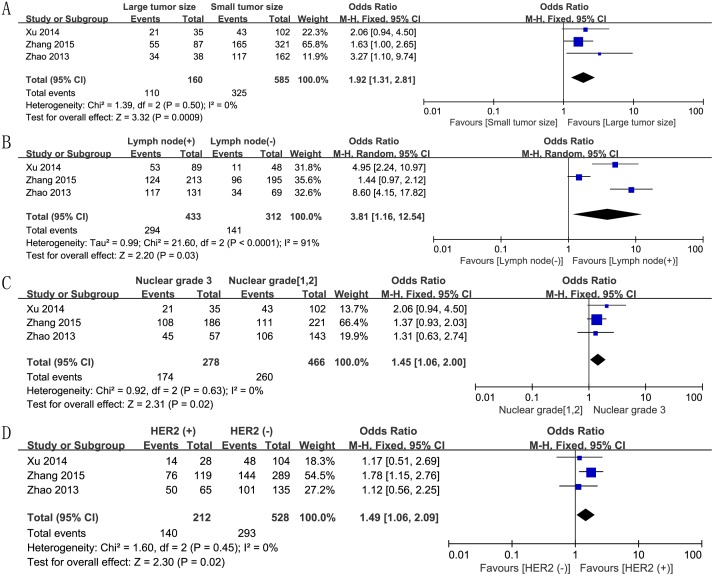
Forest plots depicting correlations between Twist expression and (A) tumor size (large *vs*. small), (B) lymph node involvement (positive *vs*. negative), (C) nuclear grade (3 *vs*. 1 and 2), and (D) HER2 status (positive *vs*. negative).

**Fig 5:**
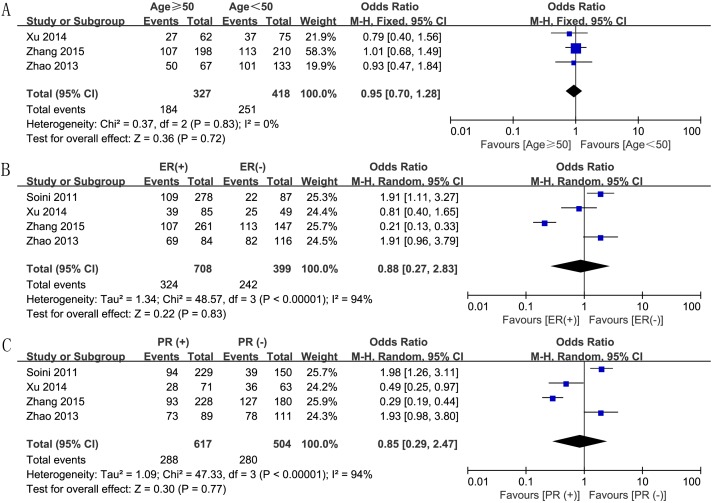
Forest plots depicting correlations between Twist expression and (A) age (≥ 50 *vs*. < 50), (B) ER status (positive *vs*. negative), and (C) PR status (positive *vs*. negative).

We also assessed the correlation between increased Twist expression and various clinical parameters of breast cancer. First, we analyzed the correlation between Twist expression and breast tumor size, based on data from 3 studies. We analyzed the data using the fixed effect model because there was no heterogeneity (P = 0.50, I^2^ = 0), and found an OR value of 1.92 (95% CI, 1.31–2.81; P = 0.0009; Fig 4A), thereby establishing a positive association of Twist expression and tumor size. The random effect model based analysis of 3 studies with high heterogeneity (P < 0.0001, I^2^ = 91%) showed significant association between Twist expression and lymph node involvement (OR, 3.81; 95% CI, 1.16–12.54; P = 0.03; Fig 4B). In addition, the fixed effect model analysis also confirmed significant association between Twist expression and increased nuclear grade (OR, 1.45; 95% CI, 1.06–2.00; P = 0.02; Fig 4C) and positive HER2 status (OR, 1.49; 95% CI, 1.06–2.09; P = 0.02; Fig 4D). However, other clinicopathological characteristics like age (OR, 0.95; 95% CI, 0.70–1.28; P = 0.72; Fig 5A), ER status (OR, 0.88; 95% CI, 0.27–2.83; P = 0.83; Fig 5B), and PR status (OR, 0.85; 95% CI, 0.29–2.47; P = 0.77; Fig 5C) did not show any association with Twist expression.

### Meta-regression analysis to identify confounding variables

We also performed meta-regression analysis to identify variable factors influencing the association of Twist expression with DFS and OS in breast cancer. However, we did not find any evidence of covariates significantly affecting DFS (S2 Table), nor did we identify any significant confounding factors as potential sources of heterogeneity in OS (S3 Table).

### Publication bias and sensitivity analysis

Our analysis of publication bias using Begg’s rank correlation test revealed no bias for DFS (P = 0.462) or OS (P = 1.000). Moreover, sensitivity analysis established that the results were stable for both DFS (S1A Fig) and OS (S1B Fig), after excluding one study at a time.

## Discussion

In our current meta-analysis, we have tried to exclusively evaluate the actual prognostic value of elevated Twist expression in breast cancer. Two earlier meta-analysis studies also tried to clarify the association of Twist expression in parallel for multiple cancers[31, 32], including breast cancer, where the results were based on data from only 2 studies. One study reported a positive association of Twist expression with worse OS in breast cancer (HR, 2.34; 95% CI, 1.72–3.20; P < 0.001)[31], while the other reported no association (HR, 1.65; 95% CI, 0.19–14.03; P = 0.66)[32]. Thus, these conflicting reports led us to undertake a comprehensive analysis. Our meta-analysis included these two studies as well as an additional five breast cancer studies to determine if there was a significant correlation between Twist expression and breast cancer. We also included studies that specifically examined Twist mRNA expression. In addition, we also examined the association between Twist expression and various breast cancer clinicopathological factors.

Interestingly, our results indicated that higher Twist expression was significantly associated with worse OS in breast cancer, but showed no correlation with DFS. This result was consistent with a previously published study by Wushou *et al*.[31], which indicated that inhibitors of Twist can be beneficial for improving clinical outcomes in breast cancer treatment. Grzegrzolka *et al*.[33] demonstrated that higher nuclear Twist expression was associated with worse event-free survival and poor OS in breast cancer patients. Lim *et al*.[34] revealed that stromal nuclear Twist over-expression was correlated with worse prognosis in terms of disease recurrence and OS in patients with phyllodes tumors of the breast. Xu *et al*.[35] indicated that increased Twist expression was related to worse distant metastasis-free survival (DMFS) in patients with breast cancer. These findings indicate that future research should focus on testing the efficiency and safety of these inhibitors. In this context, a study by Ranganathan *et al*.[36] indicated that quercetin downregulated Twist expression through inhibiting the p38MAPK pathway, resulting in breast cancer cell apoptosis. The INK4a/ARF locus was central to apoptosis through p53 pathway to inhibit proliferation[37, 38]. The earlier reports found a novel function of Twist through interfering p14ARF-mediated p53 pathway, leading to developed anti-apoptotic activity[39, 40]. Moreover, Inoue *et al*.[41] suggested that Dmp1 was a regulator of the ARF-p53 pathway. Another study by Kwilas *et al*.[42] demonstrated that a poxviral-based cancer vaccine targeting Twist suppressed breast cancer cell growth and metastasis and improved survival outcome in prostate carcinoma. Thus, these studies provided the initial evidence that there is potential benefit in targeting Twist in a therapeutic regimen for treating cancers. However, randomized controlled clinical trials are required to verify if a Twist inhibitor can really serve as a valid therapeutic strategy for cancer. Earlier literature has also reported an association between Twist over-expression and drug resistance against chemotherapeutic drugs, including Taxol in a nasopharyngeal carcinoma cell line[43], and cisplatin and doxorubicin in bladder cancer cells[44]. These observations indicate that inhibiting Twist expression could overcome chemoresistance in human tumors.

Furthermore, we also comprehensively investigated the association between elevated Twist expression and clinicopathological parameters of breast cancer. We observed that elevated Twist expression significantly correlated with larger tumor size, lymph node involvement, higher nuclear grade, and positive HER2 status. In contrast, we did not observe a significant relationship of Twist expression with age, ER status, and PR status. Previous studies have demonstrated that larger tumor size, lymph node metastasis, higher nuclear grade, and positive HER2 status are typically poor prognostic indicators of breast cancer[45–48]. Since these clinicopathological parameters showed association with higher Twist expression in our study, we conclude that our meta-analysis further validates that Twist expression is indeed associated with adverse prognosis in breast cancer. Another independent study by Vesuna *et al*.[49] reported that Twist over-expression was associated with negative ER breast cancer subtype, which is an aggressive prognostic subtype. Besides, Twist expression was notablely higher in TNBC (87.3%, 55/63), followed by the positive HER2 status (71.8%, 51/71), Luminal B (52.1%, 25/48) and Luminal A types (39.4%, 89/226) in terms of molecular subtypes in breast cancer[29] Therefore, collective observations have demonstrated that Twist over-expression could be an appropriate biomarker in breast cancer prognosis.

We should note that our study also had some limitations. First, some HRs were not offered in the original articles, and therefore HRs were extracted from the Kaplan-Meier curves for these studies. This could have impacted the robustness of outcomes. Second, each study varied with regards to Twist detection methods, as well as different variants and cut-off levels. These differences could potentially contribute to strong heterogeneity. Meta-regression analysis was performed to explore the potential sources of heterogeneity, but no significant confounding factors were observed. Finally, the sample size was also relatively small in the included studies, which could have influenced the pooled results.

In conclusion, there was evidence of a just statistically significant difference between high Twist expression and worse OS in breast cancer, however, it may be debated whether it is really clinically relevant, additional well-designed cohort studies are needed to confirm the association. Also, our study found a significant association of Twist expression with breast cancer clinicopathological characteristics, including larger tumor size, lymph node metastasis, higher nuclear grade, and positive HER2 status.

## Supporting information

S1 Fig Sensitivity analysis of Twist expression for (A) DFS and (B) OS. (TIF)

S1 Table Quality assessment of the included studies. (DOC)

S2 Table Meta-regression analysis assessing the sources of heterogeneity in DFS. (DOC)

S3 Table Meta-regression analysis assessing the sources of heterogeneity in OS. (DOC)

S1 File PRISMA 2009 checklist. (DOC)

S2 File Search strategy. (DOC)
