# Supplementary figures and images for: Prognostic and clinicopathological value of Twist expression in breast cancer: A meta-analysis
Source: PLoS One. 2017 Oct 9;12(10):e0186191. doi: 10.1371/journal.pone.0186191 (PMC5633195; doi:10.1371/journal.pone.0186191)

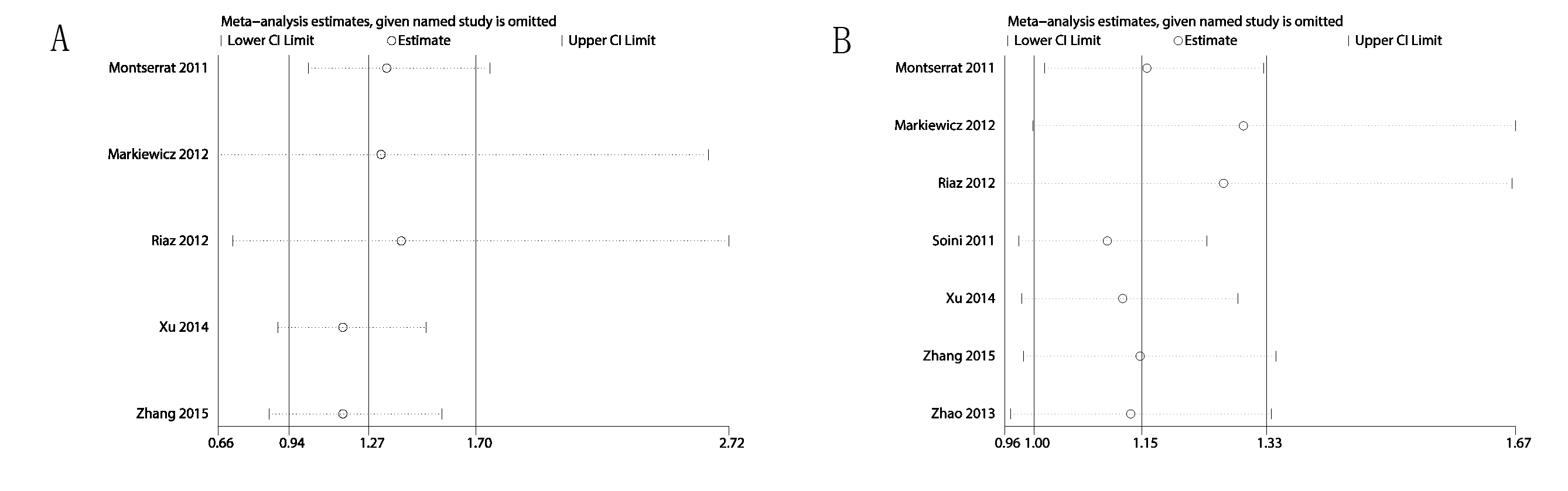

Supplement: S1 Fig — (TIF) [file pone.0186191.s001.tif]
