# Supplementary material for: Prognostic and clinicopathological value of Twist expression in breast cancer: A meta-analysis
Source: PLoS One. 2017 Oct 9;12(10):e0186191. doi: 10.1371/journal.pone.0186191 (PMC5633195; doi:10.1371/journal.pone.0186191)
Supplement: S1 Table — (DOC) [file pone.0186191.s002.doc]

S1 Table. uality assessment of the included studies.

| First author, year | Selection^1^ | | | | Comparability^2^ | Outcome^3^ | | |  |
| --- | --- | --- | --- | --- | --- | --- | --- | --- | --- |
|  | Representativeness of exposed cohort ★ | Selection of non-exposed cohort  ★ | Ascertainment of exposure ★ | No primary outcome was present at start of study ★ | Comparable  on confounder ★★ | Outcome  Assessment ★ | Adequate  follow-up★ | Loss to follow-up★ | Total Score |
| Markiewicz,2012 | ★ | ★ | ★ | ★ | ★ | ★ | ★ | ★ | 8 |
| Montserrat, 2011 | ★ | ★ | ★ | ★ | ★ | ★ | ★ | ★ | 8 |
| Riaz, 2012 | ★ | ★ | ★ |  | ★★ | ★ | ★ | ★ | 8 |
| Soini, 2011 | ★ | ★ | ★ | ★ | ★ | ★ | ★ |  | 7 |
| Xu, 2014 | ★ | ★ | ★ |  | ★★ | ★ | ★ | ★ | 8 |
| Zhang, 2015 | ★ | ★ | ★ |  | ★★ | ★ | ★ |  | 7 |
| Zhao, 2013 | ★ | ★ | ★ | ★ | ★ | ★ | ★ |  | 7 |

1“Selection” part includes representativeness of cases, selection of controls, exposure ascertainment, and no death when investigation begin.

2“Comparability” part includes comparable on confounders.

3“Outcome” part includes outcome assessment, adequate follow-up, and loss to follow-up rate.
