# Supplementary material for: Prognostic and clinicopathological value of Twist expression in breast cancer: A meta-analysis
Source: PLoS One. 2017 Oct 9;12(10):e0186191. doi: 10.1371/journal.pone.0186191 (PMC5633195; doi:10.1371/journal.pone.0186191)
Supplement: S2 Table — (DOC) [file pone.0186191.s003.doc]

S2 Table. Results of meta-regression analysis exploring the source of heterogeneity with DFS.

| Covariates | Multivariable analysis | | |
| --- | --- | --- | --- |
|  | Coefficient | SE | P value |
| Detection method | -2.25 | 0.62 | 0.17 |
| Twist phenotype | 1.66 | 0.61 | 0.23 |
| Cut-off of twist | -1.00 | 0.35 | 0.22 |
| Type of population | 0.78 | 0.42 | 0.16 |
