# Supplementary material for: Prognostic and clinicopathological value of Twist expression in breast cancer: A meta-analysis
Source: PLoS One. 2017 Oct 9;12(10):e0186191. doi: 10.1371/journal.pone.0186191 (PMC5633195; doi:10.1371/journal.pone.0186191)
Supplement: S3 Table — (DOC) [file pone.0186191.s004.doc]

S3 Table. Results of meta-regression analysis exploring the source of heterogeneity with OS.

| Covariates | Multivariable analysis | | |
| --- | --- | --- | --- |
|  | Coefficient | SE | P value |
| Detection method | -1..47 | 1.97 | 0.53 |
| Twist phenotype | 0.86 | 1.25 | 0.56 |
| Cut-off of twist | 0.25 | 0.52 | 0.97 |
| Type of population | -0.48 | 0.18 | 0.72 |
