# Supplementary material for: Prognostic and clinicopathological value of Twist expression in breast cancer: A meta-analysis
Source: PLoS One. 2017 Oct 9;12(10):e0186191. doi: 10.1371/journal.pone.0186191 (PMC5633195; doi:10.1371/journal.pone.0186191)
Supplement: S2 File — (DOC) [file pone.0186191.s006.doc]

**Pubmed:**

Search ((((Search ("Breast Neoplasms"[Mesh]) OR (((((((((((((((((((((((((((Breast Neoplasm) OR Neoplasm, Breast) OR Neoplasms, Breast) OR Tumors, Breast) OR Breast Tumors) OR Breast Tumor) OR Tumor, Breast) OR Mammary Neoplasms, Human) OR Human Mammary Neoplasm) OR Human Mammary Neoplasms) OR Neoplasm, Human Mammary) OR Neoplasms, Human Mammary) OR Mammary Neoplasm, Human) OR Mammary Carcinoma, Human) OR Carcinoma, Human Mammary) OR Carcinomas, Human Mammary) OR Human Mammary Carcinomas) OR Mammary Carcinomas, Human) OR Human Mammary Carcinoma) OR Breast Cancer) OR Cancer, Breast) OR Cancer of Breast) OR Mammary Cancer) OR Malignant Neoplasm of Breast) OR Malignant Tumor of Breast) OR Breast Carcinoma) OR Cancer of the Breast)))) AND Twist) AND ((((prognosis) OR survival) OR Prognoses) OR Prognostic Factor) Sort by: Relevance

(**165**)

**Embase:**

#1 'breast cancer'/exp OR 'breast cancer' 457,355

#2 'breast tumor' 93,139

#3 'breast carcinoma' 77,198

#4 #1 OR #2 OR #3 496,726

#5 'twist' 11,942

#6 'prognosis' 764,308

#7 'survival' 1,337,659

#8 'prognoses' 9,141

#9 'prognostic factor' 51,613

#10 #6 OR #7 OR #8 OR #9 1,861,094

#11 #4 AND #5 AND #10 375

**The Cochrane Library:**

#1 MeSH descriptor: [Breast Neoplasms] explode all trees 10162

#2 breast carcinoma:ti,ab,kw (Word variations have been searched) 2588

#3 breast cancer:ti,ab,kw (Word variations have been searched) 22847

#4 #1 or #2 or #3 24336

#5 twist:ti,ab,kw (Word variations have been searched) 278

#6 MeSH descriptor: [Prognosis] explode all trees 137738

#7 survival:ti,ab,kw (Word variations have been searched) 61718

#8 Prognoses:ti,ab,kw (Word variations have been searched) 23911

#9 Prognostic Factor:ti,ab,kw (Word variations have been searched) 7313

#10 #6 or #7 or #8 or #9 185852

#11 #4 and #5 and #10 21

**Web of Science:**

# 1 (412,604)

TOPIC: (breast cancer) OR TOPIC: (breast neoplasms) OR TOPIC: (breast carcinoma) OR TOPIC: (breast Tumor)

Indexes=SCI-EXPANDED, SSCI, A&HCI, CPCI-S, ESCI, CCR-EXPANDED, IC Timespan=All years

# 2 (54,896)

TOPIC: (twist)

Indexes=SCI-EXPANDED, SSCI, A&HCI, CPCI-S, ESCI, CCR-EXPANDED, IC Timespan=All years

# 3 (979,975)

TOPIC: (prognosis) OR TOPIC: (survival) OR TOPIC: (Prognoses) OR TOPIC: (Prognostic Factor)

Indexes=SCI-EXPANDED, SSCI, A&HCI, CPCI-S, ESCI, CCR-EXPANDED, IC Timespan=All years

# 4 (**279**)

#3 AND #2 AND #1

Indexes=SCI-EXPANDED, SSCI, A&HCI, CPCI-S, ESCI, CCR-EXPANDED, IC Timespan=All years
